# Mr. Whitewood

**Published:** 1841-12

**Authors:** 


*Mr. Whitewood.*—It is stated by Mr. George Waite, in his "Appeal," that Mr. Whitewood, formerly an eminent dental surgeon of Old Burlington street, London, went from America eighty years ago, and that he was the preceptor of his father, Mr. John Waite, by whom he was succeeded in practice.

